# Direct van der Waals Epitaxy of Crack-Free AlN Thin Film on Epitaxial WS_2_

**DOI:** 10.3390/ma11122464

**Published:** 2018-12-04

**Authors:** Yue Yin, Fang Ren, Yunyu Wang, Zhiqiang Liu, Jinping Ao, Meng Liang, Tongbo Wei, Guodong Yuan, Haiyan Ou, Jianchang Yan, Xiaoyan Yi, Junxi Wang, Jinmin Li

**Affiliations:** 1 Research and Development Center for Solid State Lighting, Institute of Semiconductors, Chinese Academy of Sciences, Beijing 100083, China; yinyue@semi.ac.cn (Y.Y.); rf@semi.ac.cn (F.R.); wyyu@semi.ac.cn (Y.W.); liangmeng@semi.ac.cn (M.L.); tbwei@semi.ac.cn (T.W.); gdyuan@semi.a.cn (G.Y.); jxwang@semi.ac.cn (J.W.); 2 Center of Materials Science and Optoelectronics Engineering, University of Chinese Academy of Sciences, Beijing 100049, China; 3 Beijing Engineering Research Center for the 3rd Generation Semiconductor Materials and Application, Beijing 100083, China; 4 Department of Electrical and Electronic Engineering, The University of Tokushima, 2-1, Minami-josanjima, Tokushima 770-8506, Japan; jpao@xidian.edu.cn; 5 Department of Photonics Engineering, Technical University of Denmark, Ørsteds Plads 345A, DK-2800 Kongens Lyngby, Denmark; haou@fotonik.dtu.dk

**Keywords:** AlN thin film, WS_2_, MOCVD, van der Waals epitaxy

## Abstract

Van der Waals epitaxy (vdWE) has drawn continuous attention, as it is unlimited by lattice-mismatch between epitaxial layers and substrates. Previous reports on the vdWE of III-nitride thin film were mainly based on two-dimensional (2D) materials by plasma pretreatment or pre-doping of other hexagonal materials. However, it is still a huge challenge for single-crystalline thin film on 2D materials without any other extra treatment or interlayer. Here, we grew high-quality single-crystalline AlN thin film on sapphire substrate with an intrinsic WS_2_ overlayer (WS_2_/sapphire) by metal-organic chemical vapor deposition, which had surface roughness and defect density similar to that grown on conventional sapphire substrates. Moreover, an AlGaN-based deep ultraviolet light emitting diode structure on WS_2_/sapphire was demonstrated. The electroluminescence (EL) performance exhibited strong emissions with a single peak at 283 nm. The wavelength of the single peak only showed a faint peak-position shift with increasing current to 80 mA, which further indicated the high quality and low stress of the AlN thin film. This work provides a promising solution for further deep-ultraviolet (DUV) light emitting electrodes (LEDs) development on 2D materials, as well as other unconventional substrates.

## 1. Introduction

Over the past few years, the van der Waals epitaxy (vdWE) of III-nitride devices has attracted a tremendous amount of attention [1,2,3,4,5,6,7]. This epitaxial mechanism is based on the weak van der Waals interaction between underlying two-dimensional (2D) materials and epitaxial layers, which will help to address the issue of lattice- and thermal-mismatch in the III-nitride heteroepitaxy [8,9]. Furthermore, semiconductors grown on 2D materials can be easily transferred to other unconventional substrates, which will create a new era for their potential applications in flexible electronics [10].

Among various 2D materials, graphene has been a focus due to the key advantage of its honeycomb crystal lattices, which are structurally compatible with the III-nitride film [7]. However, because of the lack of dangling bonds on 2D materials, the growth of high-quality III-nitride film is not an easy task. Several methods have been employed to create artificial defects, which are helpful to increase nucleation density for the subsequent growth of high-quality thin film [11]. Chung et al. conducted the growth of heteroepitaxial nitride thin film on high-density, vertically aligned ZnO nanowalls deposited on a graphene layer treated by O_2_ plasma [1]. Han et al. utilized graphene oxide microscale patterns based on sapphire substrate to realize the epitaxial lateral overgrowth of GaN [2]. Kim et al. employed the periodic nucleation sites at the step edges of graphene/SiC to realize the direct vdWE of high-quality single-crystalline GaN film [3].

AlN is the fundamental component for AlGaN-based deep-UV LEDs, which are widely applied in the field of water purification, sensing, polymerization solidification, and non-line-of-sight communication [12]. Although some progress has been made in the growth of GaN thin films on 2D materials, the growth of AlN thin films remains challenging. Our group previously reported a series of studies on the growth of AlN thin films. Qing Zeng et al. released their research into the growth of continuous AlN film on graphene, with the step edges and defects as the nucleation sites [5]. Yang Li et al. experimentally studied the feasibility of solving large mismatch problems with multilayer graphene acting as the interlayer between sapphire and the III-nitride, and further studied the effects of the optical and electrical properties of LEDs on graphene [6]. To make the action in strict van der Waals epitaxial growth on 2D materials interlayer clear, Yunyu Wang et al. investigated the roles of a graphene buffer layer in AlN nucleation on a sapphire substrate, indicating that graphene caused a decrease of nucleation density and an increase in AlN nuclei growth rate, and significantly weakened the AlN–Al_2_O_3_ interaction to release the strain [7].

The studies mentioned above are all based on the graphene buffer layer. Auxiliary methods were needed to assist the deposition of the III-nitride film (e.g., plasma treatment and ZnO nanowalls) [1,3,4]. However, the growth mechanism was changed owing to the introduction of dangling bonds, which means it is not a van der Waals epitaxy in the true sense. To realize the strict van der Waals epitaxy, more 2D materials are tested for the growth of III-nitride thin film. WS_2_ and MoS_2_ would be perfect candidates because their small lattice mismatches with III-nitrides are only 1.0% and 0.8%, respectively, to the ”a” lattice parameter of GaN [13]. In 2016, Gupta et al. proposed exhaustive studies on the growth of strain-free and single-crystal GaN islands by metal-organic chemical vapor deposition (MOCVD) on mechanically-exfoliated WS_2_ flakes [13]. Chao Zhao et al. reported the growth of InGaN/GaN nanowire LEDs on sulfurized Mo substrates [14]. Nevertheless, the growth of continuous III-nitride thin film on transition metal dichalcogenide (TMDC) buffer layers still lacks relevant research.

Motivated by these considerations, here we present the first experimental investigation of the direct epitaxy of continuous AlN thin film with a WS_2_ interlayer. Herein, high-quality AlN was obtained by MOCVD on intrinsic WS_2_/sapphire substrate. The measured root mean square (RMS) roughness was 0.230 nm. Thanks to the atomistic smoothness of the released AlN film, a fully functional 283 nm deep-ultraviolet (DUV) light emitting diodes (LEDs) device was further demonstrated.

## 2. Materials and Methods

In our work, high-purity WO_3_ (at 1000 °C) and S (at 200 °C) were applied for the synthesis of single-crystalline WS_2_ film with an area of 1 × 1 cm^2^ on sapphire substrates directly, with Ar and H_2_ as carrier gases, respectively, in a three-temperature zone tube furnace. An AlN thin film was deposited by MOCVD on the WS_2_/sapphire substrate. Trimethylaluminum (TMAl) and NH_3_ were employed as Al and N precursors, respectively. An AlN nucleation layer was first deposited at 890 °C for 4 min with a V/III ratio of about 9640. After low-temperature growth of the AlN nucleation layer, the temperature was increased to 1200 °C to grow a 500 nm AlN epilayer for 30 min with a V/III ratio of 578. No additional intermediate layers or substrate treatments were employed for AlN layer growth on WS_2_/sapphire layer. H_2_ was used as carrier gas for all of the growth steps. The MOCVD chamber pressure was kept at 50 torr during the whole growth process.

After the AlN thin film epitaxial growth, AlGaN-based DUV LED structures were further grown on the AlN/WS_2_/Sapphire template. Trimethylgallium (TMGa) was used as the Ga precursor. A 20-period AlN/Al_0.6_Ga_0.4_N superlattice (SL) was first deposited at 1130 °C, with periodic flow change of TMAl to adjust the deposition component, while the TMGa flow was kept at 32 sccm. Temperature was reduced to 1002 °C. Then, the SiH_4_ lane was opened with the flow of 20 sccm, and an n-Al_0.6_Ga_0.4_N layer was deposited with the thickness of 1.8 μm. Five-period Al_0.5_Ga_0.5_N/Al_0.6_Ga_0.4_N multiple quantum wells (MQWs) were further grown, with a 12.2 nm quantum barrier and 2.4 nm quantum well in each period. The TMAl was switched from 24 sccm to 14 sccm, while the TMGa was switched from 8 sccm to 7 sccm alternatively each time before the growth of quantum wells. A 60 nm p-Al_0.65_Ga_0.35_N electron blocking layer (EBL), a p-AlGaN cladding layer, and a p-GaN contact layer were subsequently extended. The NH_3_ was 2500 sccm during the whole growth process. After the growth, the sample were annealed at 800 °C with N_2_ flow for 20 min to activate the Mg acceptors.

Furthermore, standard LED processes were made to fabricate DUV LED, such as photolithography, ICP etching, etc. A 210 nm Ti/Al/Ti/Au metal stack and a 40 nm Ni/Au stack were respectively vapored as the n- and p-electrodes. In the end, the chips were flip-chip bonded onto ceramic submounts.

## 3. Results

**Figure 1:**
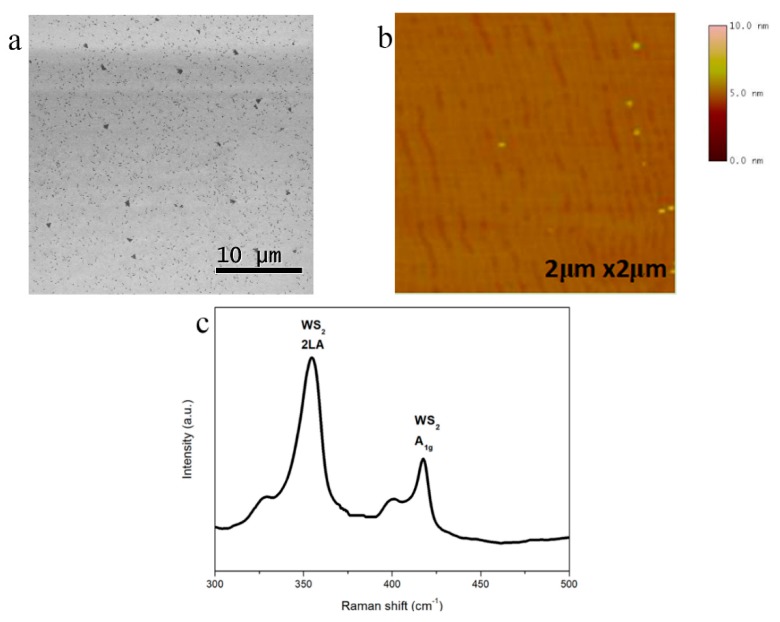
Characterizations of direct growth of WS_2_ on sapphire substrate. (**a**) Scanning electron microscopy (SEM) image of the WS_2_ film directly grown on sapphire substrates. (**b**) Atomic force microscopy (AFM) image of the WS_2_ film with root mean square (RMS) roughness around 0.203 nm. (**c**) Raman spectra of the WS_2_ film on sapphire substrates.

The surface morphology of the WS_2_ on the sapphire substrate was examined using a Hitachi S4800 scanning electron microscopy (SEM, Tokyo, Japan) operated at 3 keV acceleration voltage (Figure 1a) and tapping mode atomic force microscopy (AFM, D3100, Veeco, New York, NY, USA) (Figure 1b), indicating that the substrate could be fully covered with continuous and uniform monolayer WS_2_ film. Some secondary nuclei were attached to the WS_2_ film. A JOBIN YVON-HORIBA HR800 Raman spectrometer (Kyoto, Japan) with a semiconductor laser at 532 nm as the excitation source was employed to analyze the chemical properties and detailed compositions of the direct-grown WS_2_ film. Raman spectra presented similar intensity of 2LA and A_1g_ mode peaks (Figure 1c), which verified the good film uniformity over the area of 1 × 1 cm^2^ [13].

**Figure 2:**
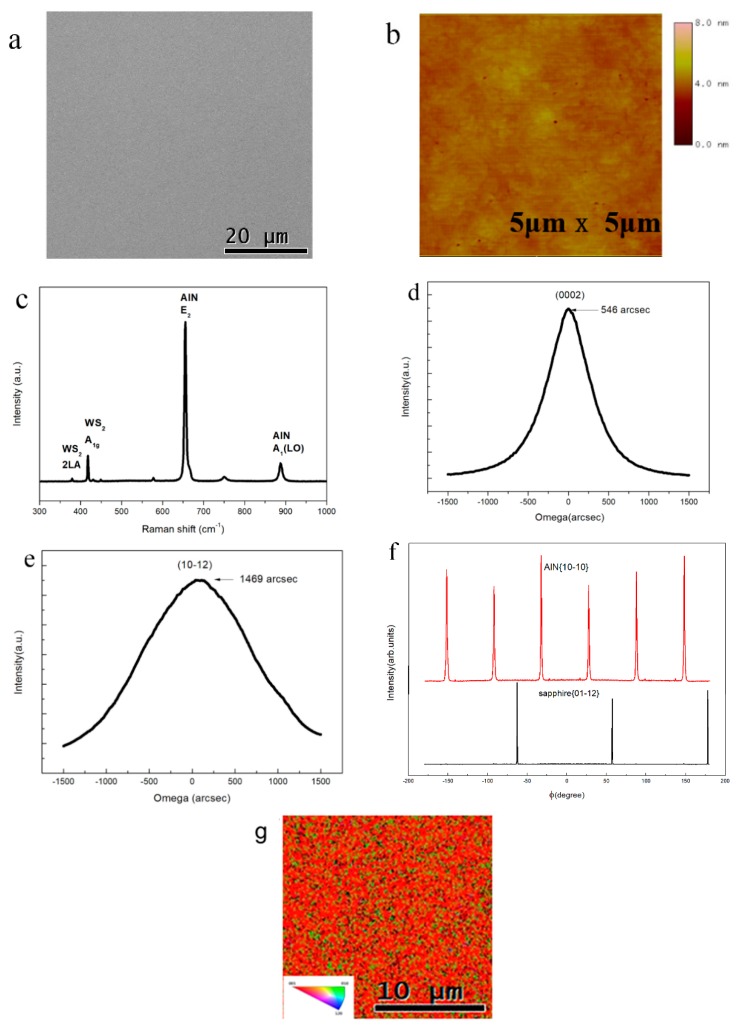
Characterizations of AlN thin film growth on WS_2_/sapphire substrate without extra treatment. (**a**) SEM image, (**b**) AFM image, (**c**) Raman spectra, (**d**) X-ray rocking curves of (0002), and (**e**) (10-12) of the AlN film grown on sapphire with WS_2_ interlayers. (**f**) X-ray powder diffraction (XRD) φ scan curve with 2θ = 25.58° χ = 57.61°. (**g**) Electron backscatter diffraction (EBSD) mapping of AlN film.

After the AlN thin film was grown on the WS_2_/sapphire substrate by MOCVD, a SEM image was obtained to investigate the surface morphology of the as-grown film, as presented in Figure 2a. Mirror-smooth and crack-free AlN thin films were grown with complete coalescence. The AFM image further verifies that the surface topology of as-grown AlN thin film on WS_2_/sapphire substrates was flat, with the RMS roughness at 0.230 nm over a lateral distance of 5 μm, as seen in Figure 2b, which was comparable with the AlN thin film directly grown on sapphire [15].

The stress of as-grown AlN thin film was further evaluated by Raman spectroscopy. The biaxial strain in the AlN layer was relative to the *E*_2_ phonon mode movement of the Raman spectrum. Figure 2c shows the AlN epilayers grown on WS_2_/sapphire substrate sustained tensile stress, demonstrating a smaller frequency (656 cm^−1^) compared with stress-free AlN (657 cm^−1^). The stress relaxation of AlN epilayers, prompted by the WS_2_ interlayer, can be appraised in light of $\Delta \omega =K\sigma_{xx}$. In this formula, Δω is the *E*_2_ peak movement between the sample and stress-free AlN crystal, while K is the biaxial stress conversion factor ≈3.7 cm^−1^·GPa^−1^ [16,17,18]. The biaxial stress value of AlN epilayers grown on WS_2_/sapphire substrate was 0.27 GPa. Compared with the Raman spectra of the WS_2_/sapphire substrate, the presence of WS_2_ after the film’s growth was confirmed by the same peak at 417.6 cm^−1^ in Figure 1c.

The crystal quality of AlN was evaluated by means of a Bede X-ray metrology double crystal high-resolution X-ray diffraction rocking curve (XRC) analyses. Figure 2d,e shows the ω-scan profiles (rocking curves) of the AlN (0002) and (10-12) peaks. The full width at half maximum (FWHM) values of the (0002) and (10-12) rocking curve of AlN are directly related to the densities of screw- and edge-dislocations in epilayers. The FWHM values of AlN thin film were measured to be 546 arcsec and 1469 arcsec, respectively, for (0002) and (10-12) reflections. The estimated densities of screw and edge dislocations were 6.49 × 10^8^ cm^−2^ and 2.42 × 10^10^ cm^−2^ [19]. Although the FWHM value was slightly larger than that of the AlN thin film grown on graphene film with extra plasma treatment. The results of rocking curves suggest the preferable c-axis alignment of the AlN film grown on the WS_2_ interlayer. To explore the epitaxial relationship between AlN epilayers and c-plane sapphire, we employed XRD-φ scan with 2θ = 25.58° χ = 57.61° (Figure 2f). Six peaks of the AlN curve could be observed. Each one was 60 degrees apart, while three peaks of the sapphire curve could be observed, and each one was 120 degrees apart. Those curves reveal that the AlN (0002) facets were rotated by 30 degrees with sapphire (0006) facets through WS_2_, describing the epitaxial relationship was [1100] AlN//[11-20] sapphire. The crystalline orientations of as-grown AlN film were also identified by using electron backscatter diffraction (EBSD). The EBSD mapping provided evidence that most of the area of the AlN film displayed almost (0001) single crystallinity, as demonstrated in red by the inverse pole figure color triangle (Figure 2g). These results all strongly suggest that single-crystalline AlN film was grown on WS_2_ film, and the DUV LEDs could be subsequently deposited on the AlN/WS_2_/sapphire template.

**Figure 3:**
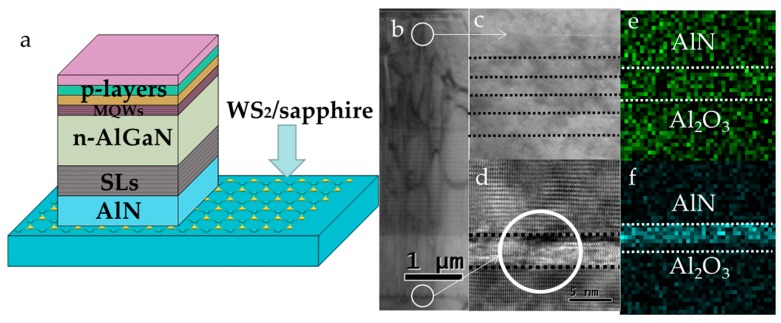
Characterizations of conventional AlGaN-based deep ultraviolet (DUV) light emitting diodes (LEDs) grown on WS_2_/sapphire substrates. (**a**) Schematic illustration of the DUV LED structure. (**b**) Cross-sectional scanning transmission electron microscopy (STEM) image of heterojunction LEDs; (**c**) Al_0.5_Ga_0.5_N/Al_0.6_Ga_0.4_N MQWs; and (**d**) the AlN/WS_2_/sapphire interface of the as-grown DUV LED. (**e**) Energy dispersive X-Ray spectroscopy (EDX) mapping of S; and (**f**) W element showing the WS_2_ gap between AlN and sapphire.

A conventional AlGaN-based DUV LED structure on WS_2_/sapphire substrate was achieved after the growth of AlN thin film. Its schematic illustration is shown in Figure 3a. In order to characterize the LED heterojunction structure and confirm the existence of WS_2_ in the AlN/WS_2_/sapphire interface, cross-sectional scanning transmission electron microscopy (STEM) and energy dispersive X-ray spectroscopy (EDX) were applied. Microstructural behaviors of the whole heterojunction grown on AlN/WS_2_/sapphire template were investigated using the cross-sectional STEM at low magnification, allowing us to scan the entire DUV LED microstructure. The cross-sectional STEM image in Figure 3b shows that layer-by-layer grown LED structures were formed, consistent with the schematic illustration. Figure 3c indicates the high quality of multiple quantum wells (MQWs) at a higher magnification, verifying that the five-period Al_0.5_Ga_0.5_N/Al_0.6_Ga_0.4_N MQWs were defect-free. Figure 3d proves the existence of WS_2_ after the growth of the LED structure. The atomically resolved STEM image shows clearly distinguishable line between AlN and sapphire as the signal of WS_2_ exists. We also investigated the existence of WS_2_ interlayers by using EDX. The elemental mapping confirmed the existence of WS_2_ interlayers with distributions of S (Figure 3e) and W (Figure 3f). W element distribution was mainly localized at the interface, with a relatively clear boundary. However, the wide distribution of S was probably the result of the decomposition of the WS_2_ layer to some extent. We tend to believe that WS_2_ layer still existed, although with many defects (e.g., S vacancy).

**Figure 4:**
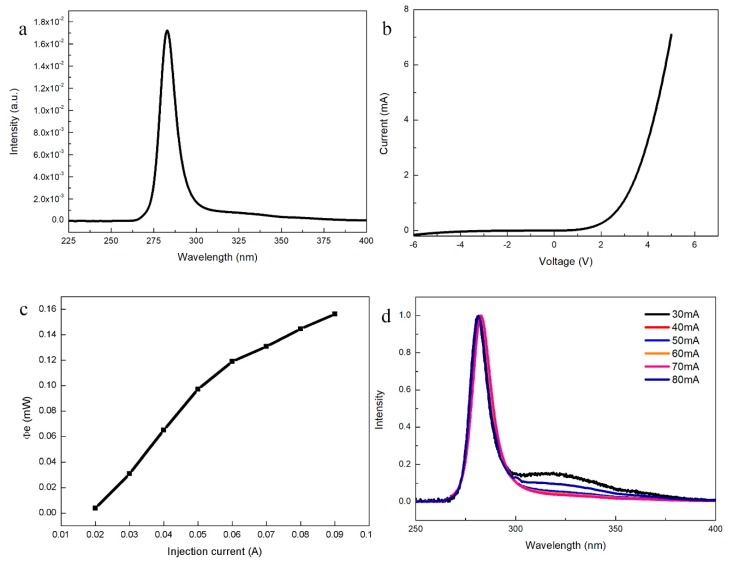
Electroluminescence (EL) of as-fabricated DUV LEDs. (**a**) The single-peaked EL spectrum of the DUV LED structure. (**b**) I-V curve of the fabricated DUV LEDs with WS_2_ buffer layer. (**c**) Light-out power (LOP) of the fabricated LEDs at various injection currents. (**d**) The normalized EL spectra of fabricated LEDs with currents ranging from 30 to 80 mA.

After the electrode deposition and other fabrication processes, the on-wafer electroluminescence (EL) performance of the DUV LED structure on the WS_2_/sapphire substrates was further investigated. A single-peaked spectrum was observed, with a peak wavelength at 283 nm at a dividing current of 80 mA (Figure 4a). Moreover, the current-voltage curve of the DUV LED with WS_2_ showed good rectifying behavior with a turn-on voltage of 3.38 V (Figure 4b), and the leakage current measured at −4 V was about 0.04 mA. This confirms that the quality of AlN thin film on WS_2_/sapphire was sufficiently robust to fabricate DUV LEDs. Figure 4c shows the functional relationship between light-out power (LOP) and injection current of LEDs. LOP increased simultaneously with the injection current, revealing that the EL emission was generated from the carrier injection and radiative recombination at MQW layers. In order to evaluate the reliability, the normalized EL of as-fabricated LED under different injection currents were investigated as shown in Figure 4d. The wavelength of the single peak only showed faint peak-position redshift from 281.8 to 283 nm, with current increasing from 30 to 70 mA, then blueshifted to 282.6 nm under the injection current of 80 mA. The inevitable thermal effect of UV devices and threading dislocation caused the redshift, while screening of the polarization electric field in strained MQW structures caused the blueshift [20,21]. The faint peak-position shift should be attributed to the low-stress property of AlN thin film. The carrier’s recombination in p-AlGaN cladding layer likely led to the weak shoulder at 324 nm with low injection current in the EL spectrum, which signifies that further optimization of the electron blocking layer to enhance the quantum confinement of electrons and suppress the electron overflow is necessary [22,23]. With increasing current, the relative intensity of the weak shoulder got weaker, until the weak shoulder vanished. These results demonstrate that conventional DUV LEDs could be fabricated on the WS_2_/sapphire substrate.

## 4. Conclusions

We demonstrated the experimental realization of crack-free and mirror-like single-crystalline AlN thin film on WS_2_ buffered sapphire substrate, resulting in RMS surface roughness of 0.230 nm, which is within the range of directly grown AlN film grown on sapphire substrate using MOCVD. The estimated densities of screw and edge dislocations were 6.49 × 10^8^ cm^−2^ and 2.42 × 10^10^ cm^−2^. Hence, the quality of AlN thin film on WS_2_/sapphire was robust enough to fabricate DUV LEDs. Fully functional DUV LED was subsequently fabricated on the AlN/WS_2_/sapphire template. Its clear EL emissions had a peak wavelength of 283 nm at 80 mA. The wavelength of the single peak only showed a faint peak-position shift with increasing current to 80 mA. The cross-sectional TEM and EDS results confirmed our growth model and the presence of the continuous WS_2_ layer in the AlN/WS_2_/sapphire hetero-interface, even after the growth of LED. The efficient DUV LEDs fabricated on WS_2_/sapphire show the potential of WS_2_ for the epitaxy of the III-nitride on large-size and low-cost metal or amorphous substrates in the future. Our work provides a potential solution for further DUV LED development on unconventional substrates.
